# Development of Chloroplast Genomic Resources for *Oryza* Species Discrimination

**DOI:** 10.3389/fpls.2017.01854

**Published:** 2017-10-25

**Authors:** Yun Song, Yan Chen, Jizhou Lv, Jin Xu, Shuifang Zhu, MingFu Li, Naizhong Chen

**Affiliations:** ^1^Institute of Plant Quarantine, Chinese Academy of Inspection and Quarantine, Beijing, China; ^2^Biological Germplasm Resources Identification Center of AQSIQ, Beijing, China; ^3^Institute of Animal Quarantine, Chinese Academy of Inspection and Quarantine, Beijing, China

**Keywords:** *Oryza*, chloroplast genome, DNA barcoding, variable markers, sequence divergence

## Abstract

Rice is the most important crop in the world as the staple food for over half of the population. The wild species of *Oryza* represent an enormous gene pool for genetic improvement of rice cultivars. Accurate and rapid identification of these species is critical for effective utilization of the wild rice germplasm. In this study, we developed valuable chloroplast molecular markers by comparing the chloroplast genomes for species identification. Four chloroplast genomes of *Oryza* were newly sequenced on the Illumina HiSeq platform and other 14 *Oryza* species chloroplast genomes from Genbank were simultaneously taken into consideration for comparative analyses. Among 18 *Oryza* chloroplast genomes, five variable regions (*rps16-trnQ, trnTEYD, psbE-petL, rpoC2* and *rbcL-accD*) were detected for DNA barcodes, in addition to differences in simple sequence repeats (SSR) and repeat sequences. The highest species resolution (72.22%) was provided by *rpoC2* and *rbcL-accD* with distance-based methods. Three-marker combinations (*rps16-trnQ* + *trnTEYD* + *rbcL-accD, rps16-trnQ* + *trnTEYD* + *rpoC2* and *rpoC2 + trnTEYD + psbE-petL*) showed the best species resolution (100%). Phylogenetic analysis based on the chloroplast genome provided the best resolution of *Oryza*. In the comparison of chloroplast genomes in this study, identification of the most variable regions and assessment of the focal regions of divergence were efficient in developing species-specific DNA barcodes. Based on evaluation of the chloroplast genomic resources, we conclude that chloroplast genome sequences are a reliable and valuable molecular marker for exploring the wild rice genetic resource in rice improvement.

## Introduction

Rice is an important cereal crop that provides essential food and energy for more than half of the world’s population. The genus *Oryza* has two cultivated species (*O. sativa* and *O. glaberrima*) and approximately 21 species of wild relatives. Rice with an AA genome type is the most important species, which has two subspecies with global distributions, *O. sativa* ssp. *japonica* and *O. sativa* ssp. *indica*. The genus *Oryza* is classified into 10 genome types, including six diploids (AA, BB, CC, EE, FF, and GG) and four allotetraploids (BBCC, CCDD, HHJJ, and HHKK) (Aggarwal et al., 1999).

The wild species contain valuable genetic diversity that has continued to contribute immensely to rice crop improvement (Choi et al., 2017). Because of the importance of *Oryza* as a major food crop, *Oryza* species have been the subject of studies on taxonomy, phylogenetic relationships of species within the genus, and species identification (Zou et al., 2008; Tang et al., 2010; Zhang et al., 2014). Such approaches provide tremendous benefits in plant breeding and to effective conservation. For the effective exploration, conservation, and domestication, accurately identified wild species would provide clarity. However, taxonomy of wild species remains problematic. For example, the A-genome group, also called the *Oryza sativa* complex, consisting of eight diploid species, is particularly problematic because most of them lack clear morphological distinguishing characteristics (Vaughan, 1989; Zhu and Ge, 2005).

Biological specimens are identified using morphological features, and in most cases, an experienced professional taxonomist is required. Molecular marker methods and DNA barcoding, offer effective information for taxonomy and species discrimination. In recent decades, the applications of various molecular techniques (SSR, AFLP, and DNA barcodes, among others) have played important roles in resolving questions concerning taxonomy and species discrimination. DNA barcoding can provide a new tool for accurate species discrimination (Hebert et al., 2003). DNA barcoding involves sequencing one or more standard genomic regions as a tool for species identification. Recently, a two-locus land plant barcode consisting of portions of the chloroplast genes *rbcL* and *matK* or *ycf1b* and the nuclear gene ITS have been proposed (CBOL Plant Working Group, 2009).

For phylogenetic studies of *Oryza*, molecular markers, including chloroplast genome regions (Ge et al., 1999; Tang et al., 2010), complete chloroplast genomes (Wambugu et al., 2015; Tong et al., 2016), multiple nuclear genes (Zou et al., 2008), and SNPs (Wang et al., 2017) have been analyzed. However, an effective DNA barcode for *Oryza* remains unclear. One source of sequence of diversity that can be used to identify species is the chloroplast genome. The chloroplast genome has a highly conserved circular DNA arrangement ranging from 115 to 165 kb, with 130 genes encoded. Chloroplast genomes have often been used for phylogenetic studies and species identification because of the slower evolution than that of nuclear genomes. Moreover, in most angiosperms, genetic information from the chloroplast genome is inherited maternally, with the chloroplast genome then a good indicator of maternal ancestry. Most studies show that chloroplast genome mutations are not random but clustered as “hot spots” (Dong et al., 2012, 2014a; Song et al., 2015; Xu et al., 2017), and this mutation dynamic has created highly variable regions in the genome.

With the recent availability of the next-generation sequencing techniques, much sequence data can be generated at relatively low cost with time saved. In this study, we sequenced and analyzed the chloroplast genome of *O. meyeriana, O. latifolia, O. eichingeri*, and *O. rhizomasis* using the Illumina HiSeq platform. The first objective was to obtain useful chloroplast molecular markers by comparing the chloroplast genomes for species identification. The second objective was to evaluate the interspecific variation within the genus. The third objective was to reveal the structural patterns of the *Oryza* chloroplast genome.

## Materials and Methods

### Taxon Sampling, DNA Extraction, and Sequencing

Fresh leaves of the four species of *Oryza* were collected from the Kunming Institute of Botany, Chinese Academy of Sciences and the Rice Research Institute, Guangdong Academy of Agricultural Sciences. Fresh leaves from each accession were immediately dried with silica gel before DNA extraction. Total genomic DNA was extracted and purified following the method of Li et al. (2013). DNA samples were randomly fragmented into 400–600 bp fragments using an ultrasonicator. An Illumina paired-end DNA library with 500-bp insert size was constructed using a NEBNext^®^ Ultra^TM^DNA Library Prep Kit following the manufacturer’s instructions. Paired-end sequencing (2 × 150 bp) was conducted on an Illumina HiSeq X Ten platform.

### Genome Assembly and Genome Annotation

The paired-end reads were qualitatively assessed and assembled with SPAdes 3.6.1 (Bankevich et al., 2012). Chloroplast genome sequence contigs were selected from SPAdes software by performing a BLAST search using the *Oryza sativa* chloroplast genome sequence as a reference (GenBank accession number: JN861110). The selected contigs were assembled with Sequencher 5.4.5 (Gene Codes, Ann Arbor, MI, United States). Small gaps in the assemblies were bridged with specific primers designed for PCR based on their flanking sequences and then by Sanger sequencing. Based on the reference chloroplast genome, the four junctions between the inverted repeats (IRs) and single-copy regions were checked by amplification with specific primers followed by Sanger sequencing (Dong et al., 2013). Chloroplast genome annotation was performed with Plann (Huang and Cronk, 2015) using the *Oryza sativa* reference sequence from Genbank. A chloroplast genome map was drawn using Genome Vx software (Conant and Wolfe, 2008).

### Analysis of Tandem Repeats and Single Sequence Repeats

MIcroSAtellite (MISA^1^) was used to detect single sequence repeats (SSR) within the chloroplast genome, with the parameters set at >10 for mononucleotide, >5 for dinucleotide, >4 for trinucleotide, and >3 for tetranucleotide, pentanucleotide, and hexanucleotide SSRs. The web-based software REPuter was used to visualize the dispersed repeat sequences in *Oryza* by forward vs. reverse complement (palindromic) alignment (Kurtz et al., 2001). The following settings for repeat identification were used: (1) a minimum repeat size of 30 bp; (2) Hamming distance of 3; and (3) 90% or greater sequences identity. Tandem repeats were identified using web-based Tandem Repeats Finder^2^, with 2, 7, and 7 set for the alignment parameters match, mismatch, and indel, respectively.

### Sequence Divergence Analysis and Phylogenetic Analyses

Complete chloroplast genomes were used to analyze the average pairwise sequence divergence for the 18 *Oryza* species (**Table 1**). All *Oryza* sequenced chloroplast genomes were aligned using MAFFT v7 (Katoh and Standley, 2013) assuming collinear genomes for the full alignment and then adjusted manually using Se-Al 2.0 (Rambaut, 1996). Variable and parsimony-informative base sites were calculated using MEGA 6.0 software (Tamura et al., 2013). The p-distances among *Oryza* chloroplast genomes were calculated to evaluate the divergence of *Oryza* species using MEGA software.

**Table 1:** A list of the 14 taxa sampled from Genbank in this study.

Species	Accession number in Genbank
*Oryza australiensis*	KJ830774
*Oryza barthii*	KM103379
*Oryza brachyantha*	KT992850
*Oryza glaberrima*	KM881638
*Oryza glumipatula*	KM881640
*Oryza longistaminata*	KM881642
*Oryza meridionalis*	KM103373
*Oryza minuta*	KU179220
*Oryza nivara*	KM088022
*Oryza officinalis*	KM881643
*Oryza punctata*	KM103375
*Oryza rufipogon*	KF562709
*Oryza sativa Indica*	JN861110
*Oryza sativa Japonica*	KM088016

Phylogenetic trees were constructed by maximum parsimony (MP), maximum likelihood (ML), and Bayesian inference (BI) using the entire chloroplast genome. The chloroplast genome sequences of *Leersia tisserantii, Zizania latifolia, Zizania aquatic, Rhynchoryza subulata, Chikusichloa aquatic, Potamophila parviflora*, and *Microlaena stipoides* were used as out-groups.

Maximum parsimony analyses were conducted using PAUP v4b10, which was performed using heuristic searches with the ‘MulTrees’ option followed by tree bisection–reconnection (TBR) branch swapping. To assess node support, bootstrap analyses were performed using 1000 replicates with 10 random taxon additions and heuristic search options. All characters were equally weighted, gaps were treated as missing, and character states were treated as unordered. ML analyses were performed using RAxML v.8.1.24 with general time reversible (GTR) + G model. Supports for nodes were assessed with 1,000 rapid bootstrapping replicates. BI was conducted with Mrbayes v3.2 (Ronquist et al., 2012). The analysis was run for 1,000,000 generations and sampled every 1,000 generations. The first 25% of the trees were discarded as burn-in, the remaining trees were used to build a 50% majority-rule consensus tree and estimate the Bayesian posterior probabilities.

### Divergent Hot Spot Identification

Hypervariable chloroplast regions were identified using *slideAnalyses* of SPIDER (Brown et al., 2012) version 1.2-0. This function extracts all the passable windows of a chosen size in a DNA alignment and performs pairwise distance (K2P) analyses of each window. Two factors were considered for the definition of hypervariable regions: first, the mean distance of each window, and second, the proportion of zero pairwise distances for each species in the matrix. The step size was set to 200 bp, with an 800-bp window length. Two data sets were created for this analysis: (1) all 18 species data set and (2) only AA genome species data set (included nine species).

### DNA Barcoding Analysis

We analyzed the hypervariable barcodes and compared the chloroplast genes *rbcL, matK*, and *trnH-psbA* using two different methods. To assess the barcoding resolution for all barcodes, the distance and the tree-building methods were the two analytical approaches. Distance may be the most commonly used method for DNA sequences classification, which was used to evaluate the barcode performances of the newly identified highly variable regions. The function *nearNeighbour* of SPIDER was used for barcoding analyses (Brown et al., 2012).

Tree building analyses provide a convenient and visualized method for evaluating discriminatory performance by calculating the proportion of monophyletic species. MP trees were constructed for each hypervariable marker and the different marker combinations using MEGA 6.0. Relative support for the branches of the MP tree was assessed via 1,000 bootstrap replicates. Species were considered successfully discriminated when all individuals of a species formed a single and exclusive clade in a MP tree with bootstrap value above 50%.

## Results

### Chloroplast Genome Generation, Characterization, and Annotation

Using an Illumina HiSeq X ten system, the four species of *Oryza* were sequenced, producing from 9,642,763 to 10,287,100 paired-end raw reads (150 bp for average read length). After mapping the paired-end reads with the reference chloroplast genome of *Oryza sativa*, 105,824 to 499,727 reads were extracted, with 117× to 556× coverage (**Table 2**). PCR-based sequencing validated four junction regions in each of the *Oryza* chloroplast genomes. The four *Oryza* chloroplast genome sequences were deposited in GenBank (accession numbers, MF401450–MF401453).

**Table 2:** Summary statistics for assembly of four *Oryza* species chloroplast genomes.

Gene features	*Oryza meyeriana*	*Oryza latifolia*	*Oryza eichingeri*	*Oryza rhizomasis*
Raw data no.	9,642,763	10,287,100	9,797,240	9,910,302
Mapped read no.	105,824	140,832	211,039	499,727
Mapped to reference genome (%)	1.10	1.37	2.15	5.04
Chloroplast genome coverage (×)	117	156	235	556
Size (bp)	135,236	135,191	134,817	134,748
LSC length (bp)	81,135	81,212	80,844	80,788
IR length (bp)	20,802	20,820	20,822	20,817
SSC length (bp)	12,497	12,339	12,329	12,326
Number of genes	110	110	110	110
Protein coding genes	77	77	77	77
tRNA genes	29	29	29	29
rRNA genes	4	4	4	4
GC content (%)	39.1	39.0	39.0	39.0
Accession number in Genbank	MF401453	MF401451	MF401450	MF401452

**FIGURE 1:**
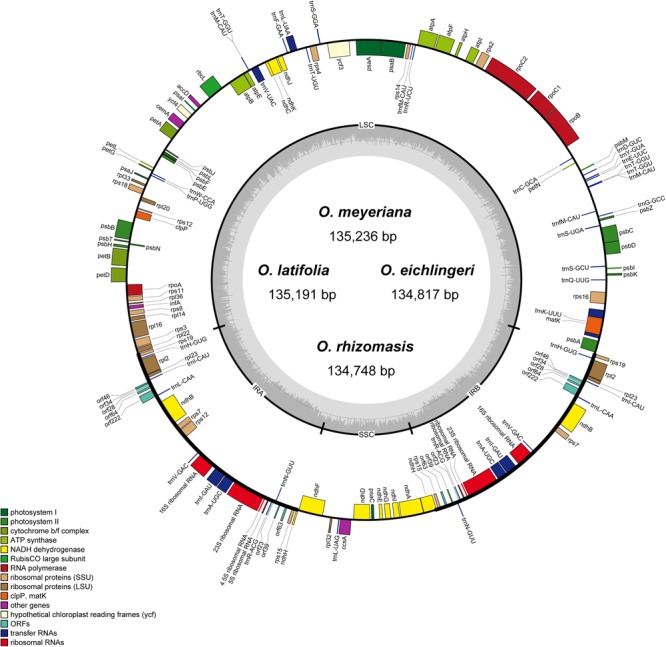
Gene map of *Oryza* chloroplast genome. Genes on the inside are transcribed in the clockwise direction while genes on the outside are transcribed in the counterclockwise direction. Genes in different functional groups are shown in different colors. The thick lines indicate the extent of the inverted repeats (IRa and IRb).

The chloroplast genome of the four species of *Oryza* ranged from 134,748 bp (*O. rhizomasis*) to 135,239 bp (*O. meyeriana*). Chloroplast genomes showed a typical quadripartite structure, consisting of a pair of IRs (20,817–20,822 bp) separated by the LSC (80,788–81,212 bp) and SSC (12,326–12,497 bp) regions (**Figure 1** and **Table 2**). For all four chloroplast genomes, the average GC content was 39.0%. The annotation chloroplast genomes of these four species were represented in one circular map, because their gene number, order and names were the same. With the duplicated genes in IR regions only counted once, the chloroplast genomes of the four species of *Oryza* harbored 110 different genes, including 77 protein-coding genes, 29 tRNA genes and 4 rRNA genes. The gene number and the genome organization are very similar to the chloroplast genomes of other *Oryza* species.

### SSR and Repeat Analyses

**FIGURE 2:**
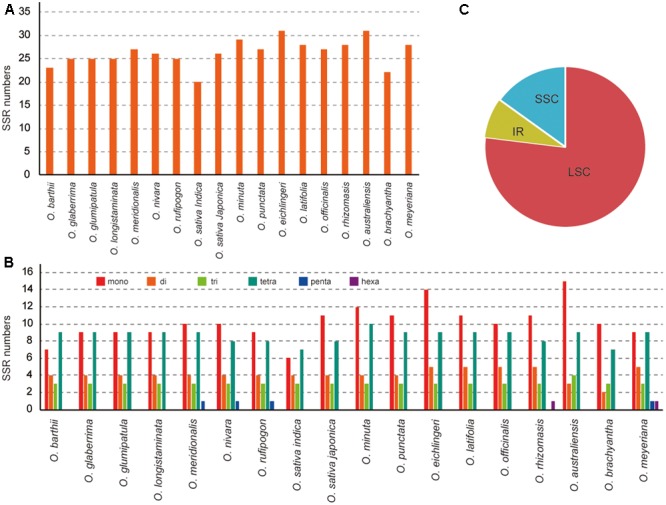
Analysis of perfect simple sequence repeats (SSRs) in 18 *Oryza* chloroplast genomes. **(A)** Number of SSRs detected in 18 chloroplast genomes. **(B)** Frequency of identified SSRs in LSC, IR, and SSC regions. **(C)** Number of SSR types detected in 18 chloroplast genomes.

In this study, we detected SSRs and dispersed repeats in all published *Oryza* species chloroplast genomes. Each *Oryza* chloroplast genome contained 22–31 SSRs (**Figure 2**). Among those SSRs, most were located in the LSC/SSC regions (92.0%). For each species, mono-, di-, tri-, tetra-, penta-, and hexanucleotide SSRs were all detected, with the average of mono-, di-, tri-, and tetranucleotide SSRs 38.69%, 15.64%, 11.63%, and 32.77%, respectively. Additionally, we did not find many penta- and hexanucleotide SSRs in *Oryza*, which is similar to the chloroplast genome of angiosperm species (Choi et al., 2016; Xu et al., 2017). SSRs were particularly rich in AT in the *Oryza* chloroplast genomes. The majority of SSRs in all species were A/T mononucleotides.

**FIGURE 3:**
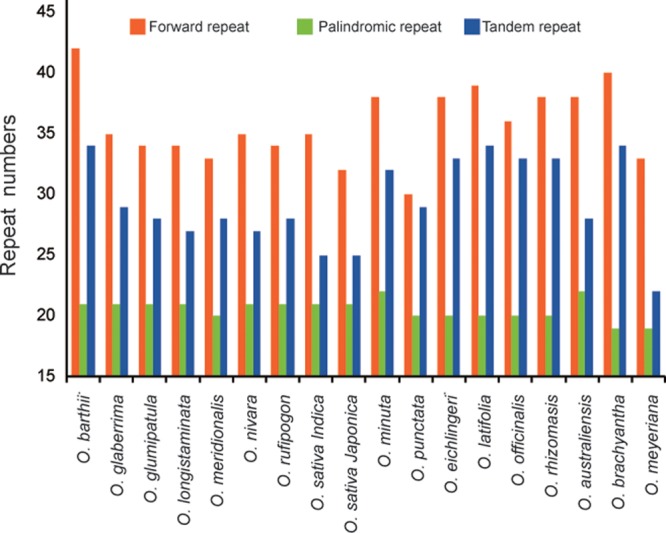
Analysis of repeated sequences in 18 *Oryza* chloroplast genomes.

Dispersed repeat sequences, which play a role in genome rearrangement, have been used as a source to understand the phylogenetic relationships of species. Repeat sequences with a repeat unit longer than 30 bp were analyzed (**Figure 3**). Each *Oryza* chloroplast genome contained 50–63 repeat sequences, including 30–42 forward repeats, 19–22 palindromic repeats, and 22–34 tandem repeats. The repeats were primarily located in non-coding regions of chloroplast genomes.

### Genome Sequence Divergence

Eighteen *Oryza* chloroplast genomes were fully aligned, and the alignment matrix of 139,988 bp was obtained. The alignment revealed high sequence similarity across the *Oryza* chloroplast genomes, suggesting that they are highly conserved. We retrieved 3,593 variable sites (2.57%), including 2,017 singletons and 1,576 parsimony-informative sites (1.13%), in the total chloroplast genome.

The number of nucleotide substitutions and p-distance were used to estimate the divergence among the eighteen *Oryza* species. The number of nucleotide substitutions among seven species varied from 13 to 1,953, and the p-distance varied from 0.0001 to 0.0149 (**Supplementary Table S1**). The overall sequence divergence estimated by p-distance among the four genomes was only 0.0052. The largest sequence divergence was observed between *O. minuta* and *O. brachyantha* and the lowest divergence was between *O. barthii* and *O. glaberrima.* At the level of the genome type, the largest sequence divergence occurred between the FF genome group and AA or BB genome group (**Supplementary Table S2**), with the sequence distance (0.0147). The smallest sequence divergence was observed between the CC genome group and CCDD genome group, with the sequence distance (0.0012).

### Phylogenetic Analyses

**FIGURE 4:**
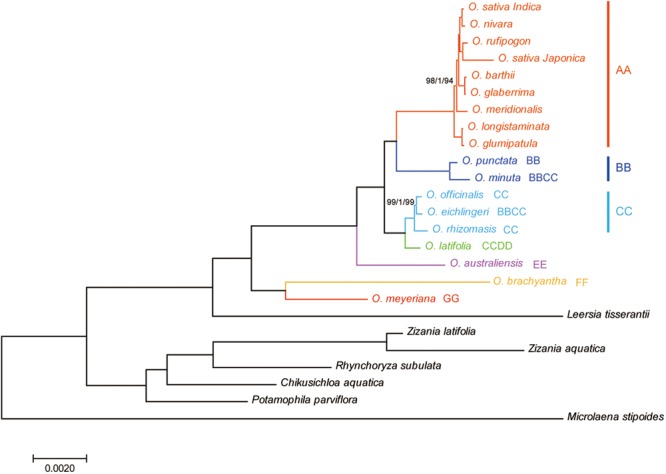
Phylogenetic tree reconstruction of 25 taxa using maximum likelihood, Bayesian inference, and maximum parsimony methods based on the complete chloroplast genome sequences. ML topology shown with ML bootstrap support value/Bayesian posterior probability/MP bootstrap support given at each node. Nodes with 100 ML-BP/1.0 BI-PP/100 MP-BP are not marked.

Phylogenetic relationships within the *Oryza* were reconstructed using MP, ML, and BI analyses. The topologies based on the three methods were highly supported and largely congruent. **Figure 4** illustrates the phylogeny generated by the ML analysis, including three types of support values: MP bootstrap values (MP-BS), ML bootstrap values (ML-BS), and BI posterior probabilities (BI-PP). All analyses fully supported the monophyly of the genus *Oryza* (ML-BS/BI-PP/MP-BS = 100/1.0/100). Within *Oryza*, the eighteen species divided into seven major groups with different genome types (AA, BB, CC, CCDD, EE, FF, and GG). The CCDD genome species formed a monophyletic clade with the CC genome, which suggested that the CC genome served as the maternal parent of the CCDD genome species. Two BBCC genome species, *O. minuta* and *O. eichingeri*, had different maternal origins, with a maternal parent of the BB genome for *O. minuta* and a maternal parent of the CC genome for *O. eichingeri*. *O. longistaminata*, and *O. glumaepatula* formed the basal clade in the AA group, which are found in Africa and South America, respectively.

### DNA Barcode Development and DNA Barcoding Analysis

**FIGURE 5:**
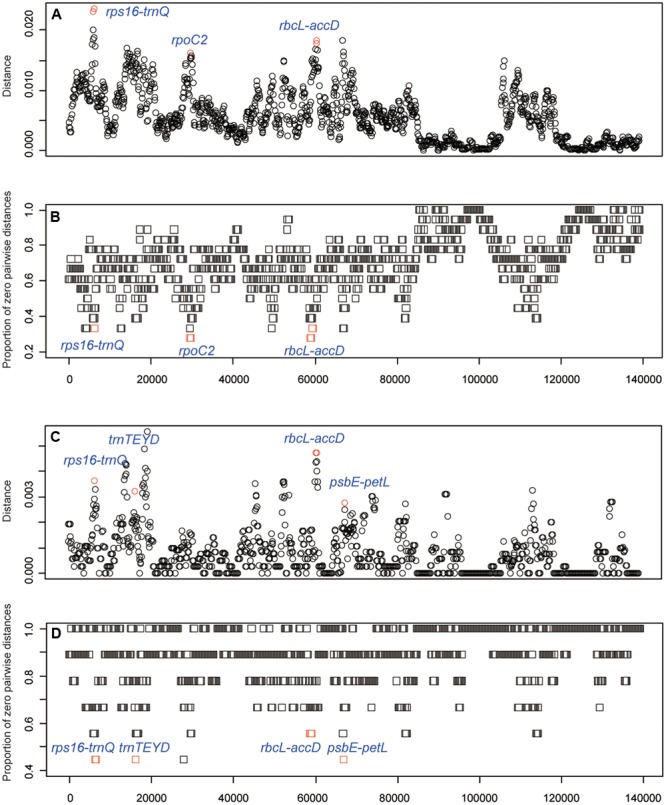
DNA barcode development. **(A)** Mean distance of each window using the 18 species data set. **(B)** The proportion of zero pairwise distances for each species based on the 18 species data set. **(C)** Mean distance of each window using the AA genome species data set. **(D)** The proportion of zero pairwise distances for each species based on the AA genome species data set.

To identify the hypervariable chloroplast regions, pairwise distance values within 800 bp in the eighteen *Oryza* chloroplast genomes were calculated with *slideAnalyses* of SPIDER (**Figure 5**). Three regions showed remarkably high distances and low proportions of zero pairwise distances for each species in the 18 species data set. One region was the coding region of *rpoC2* and two were the intergenic regions (*rps16-trnQ* and *rbcL-accD*). For the data set of AA genome species, four variable chloroplast regions (*rps16-trnQ, trnTEYD, psbE-petL* and *rbcL-accD*) were identified.

The variability of the five developed variable regions was tested together with three conventional candidate DNA barcodes (*matK, rbcL* and *trnH-psbA*). Features of the eight barcode data set are shown in **Table 3**. The conventional candidate DNA barcodes had lower variability than that of the newly developed markers. The highest variability was in the *rpoC2* region (6.80%), followed by the regions *psbE-petL* region (6.75%), *rps16-trnQ* (6.73%), *trnTEYD* (6.00%), and *rbcL-accD* (5.22%).

**Table 3:** Variability of the five new markers and the universal chloroplast DNA barcodes in *Oryza.*

Markers	Length	Variable sites		Information sites		Discrimination success (%) based on Distance method
		Numbers	%	Numbers	%	
*rps16-trnQ*	1,500	101	6.73%	45	3.00%	66.67%
*rpoC2*	1,000	68	6.80%	37	3.70%	72.22%
*rbcL-accD*	900	47	5.22%	16	1.78%	72.22%
*trnTEYD*	800	48	6.00%	22	2.75%	50.00%
*psbE-petL*	800	54	6.75%	28	3.50%	66.67%
*rps16-trnQ + trnTEYD*	2,300	149	6.48%	67	2.91%	88.89%
*rps16-trnQ + psbE-petL*	2,300	155	6.74%	73	3.17%	77.78%
*rpoC2 + trnTEYD*	1,800	116	6.44%	59	3.28%	88.89%
*rpoC2 + psbE-petL*	1,800	122	6.78%	65	3.61%	94.44%
*rbcL-accD + trnTEYD*	1,700	95	5.59%	38	2.24%	83.33%
*rbcL-accD + psbE-petL*	1,700	101	5.94%	44	2.59%	83.33%
*rps16-trnQ + rbcL-accD*	2,400	148	6.17%	61	2.54%	88.89%
*rps16-trnQ + rpoC2*	2,500	169	6.76%	82	3.28%	83.33%
*rpoC2 + rbcL-accD*	1,900	115	6.05%	53	2.79%	83.33%
*trnTEYD + psbE-petL*	1,600	102	6.38%	50	3.13%	77.78%
*rps16-trnQ + rbcL-accD + trnTEYD*	3,200	196	6.13%	83	2.59%	100.00%
*rpoC2 + trnTEYD + psbE-petL*	2,600	170	6.54%	87	3.35%	100.00%
*rps16-trnQ + rpoC2 + trnTEYD*	3,300	217	6.58%	104	3.15%	100.00%
*rbcL*	800	22	2.75%	12	1.50%	33.33%
*matK*	818	41	5.01%	26	3.18%	33.33%
*trnH-psbA*	563	12	2.13%	9	1.60%	16.67%
*rbcL + matK*	1,618	63	3.89%	38	2.35%	33.33%
*rbcL + matK + trnH-psbA*	2,181	75	3.44%	47	2.15%	38.89%

**FIGURE 6:**
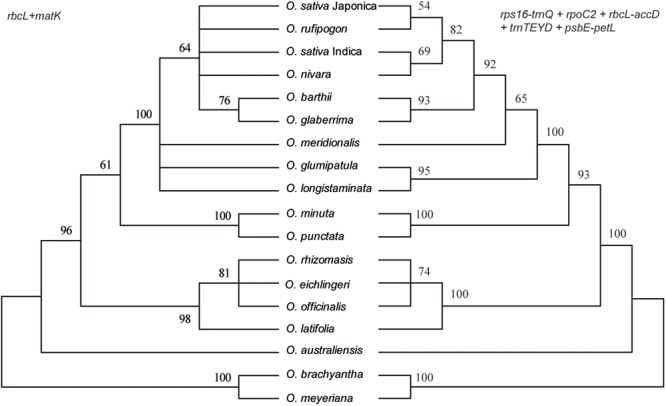
MP tree for *Oryza* using the *rbcL + matK* and *rps16-trnQ + rpoC2 + rbcL-accD + trnTEYD + psbE-petL* DNA barcode combinations.

Of the single-region barcodes, *rpoC2* and *rbcL-accD* had the highest rate of correct identifications (72.22%), followed by *rps16-trnQ* and *psbE-petL* (66.67%) and *trnTEYD* (50.00%). The conventional candidate DNA barcodes had less discriminatory power, e.g., *trnH-psbA* had only a 16.67% success rate. Of the two-region barcodes, the best performing was *rpoC2 + psbE-petL* (94.44%). With the two core DNA barcodes *rbcL* and *matK* combined, success was only 33.33%. When analyzing multi-region barcodes, the highest correct identifications (100%) were with *rps16-trnQ* + *trnTEYD* + *rbcL-accD, rps16-trnQ* + *trnTEYD* + *rpoC2*, and *rpoC2 + trnTEYD + psbE-petL*. The MP tree method generated a graphical representation of the results, and the results of the tree-based method were the same (**Figure 6**).

## Discussion

Knowing the name of an organism is essential, because the name is the same anywhere, indicating a specific organism, whether in medicine, seed, or wood form. However, the accurate discrimination of material using only morphological characteristics is difficult. DNA barcoding has become conventional technology used in discrimination. Although DNA barcoding technology has developed significantly, no barcode achieves the goal of sophisticated identification of plant species. In fact, the identification of species with short evolutionary times and close genetic relationships continues to face great challenges (Fazekas et al., 2008).

The ideal DNA barcode would be a single locus that could be universally amplified and sequenced for a broad range of taxa, easily aligned over large phylogenetic distances and that provided sufficient variation to reliably distinguish closely related species (Clement and Donoghue, 2012). Unfortunately, the candidate barcodes such as *matK* and *rbcL*, as “core” plant barcodes, perform well in terms of universality and/or sequence quality but often have limited resolutions at the species level (Dong et al., 2015). In this study, the combination of *matK, rbcL*, and *trnH-psbA* have poor resolution within *Oryza*, only less than half of the samples were successfully identified within *Oryza* (**Table 3** and **Figure 6**). Therefore, development of effective, specific barcodes with high identification rates is very important for *Oryza*.

The chloroplast genome has additional effective resources for the development of specific barcodes (Dong et al., 2012, 2014b; Song et al., 2017; Xu et al., 2017). Some “hotspot” regions occur in the chloroplast genome that evolve very rapidly and meet the criteria required to be a DNA barcode. The strategy of searching the whole chloroplast genome has been successfully applied to *Panax* (Dong et al., 2014b), *Paris* (Song et al., 2017), *Quercus* (Yang et al., 2017), and *Lagerstroemia* (Xu et al., 2017). Therefore, the strategy to identify the best chloroplast DNA barcodes is reliable. In this study in the comparison of the eighteen *Oryza* chloroplast genomes, we identified five highly variable regions (candidate DNA barcodes), including *rps16-trnQ, trnTEYD, psbE-petL, rpoC2*, and *rbcL-accD* (**Figure 5**). These markers provided effective resolution of *Oryza*. The regions *rps16-trnQ* and *rbcL-accD* have been the focus in previous studies assessing DNA barcodes in angiosperms (Dong et al., 2012) or as species-specific DNA barcodes (Xu et al., 2017); however, *trnTEYD, psbE-petL*, and *rpoC2* are rarely reported.

## Conclusion

In this study, we generated chloroplast genomes for four *Oryza* species using Illumina HiSeq platforms and compared those genomes with the other published *Oryza* chloroplast genomes. The complete chloroplast genome of *Oryza* species had conserved genome structures and size and gene contents. The chloroplast genomes provided sufficient genetic information for species discrimination and hypervariable regions have been identified by comparing the chloroplast genomes. The specific *Oryza* DNA barcodes were tested and found useful for identifying *Oryza* species.

## Author Contributions

YS and JL designed the experiment, drafted the manuscript. YC collected samples and performed the experiment. YS and JX analyzed the data. NC, ML, and SZ contributed reagents and analysis tools. All of the authors have read and approved the final manuscript.

## Conflict of Interest Statement

The authors declare that the research was conducted in the absence of any commercial or financial relationships that could be construed as a potential conflict of interest.

## Footnotes

1 http://pgrc.ipk-gatersleben.de/misa

2 https://tandem.bu.edu/trf/trf.html

## Supplementary Material

TABLE S1 Numbers of nucleotide substitutions and sequence distances in 18 complete chloroplast genomes. The upper triangle shows the number of nucleotide substitutions. The lower triangle indicates the number of sequence distances in complete chloroplast genomes.

TABLE S2 Sequence distances among the seven *Oryza* groups.
